# Nonlinear optical components for all-optical probabilistic graphical model

**DOI:** 10.1038/s41467-018-04578-x

**Published:** 2018-05-29

**Authors:** Masoud Babaeian, Pierre-A. Blanche, Robert A. Norwood, Tommi Kaplas, Patrick Keiffer, Yuri Svirko, Taylor G. Allen, Vincent W. Chen, San-Hui Chi, Joseph W. Perry, Seth R. Marder, Mark A. Neifeld, N. Peyghambarian

**Affiliations:** 1 Department of Physics, University of Arizona, Tucson, AZ 85721 USA; 2 College of Optical Sciences, University of Arizona, Tucson, AZ 85721 USA; 3 Institute of Photonics, University of Eastern Finland, Joensuu, FI 80101 Finland; 4 School of Chemistry and Biochemistry, Georgia Institute of Technology, Atlanta, GA 30332 USA; 5 Electrical and Computer Engineering, University of Arizona, Tucson, AZ 85721 USA

## Abstract

The probabilistic graphical models (PGMs) are tools that are used to compute probability distributions over large and complex interacting variables. They have applications in social networks, speech recognition, artificial intelligence, machine learning, and many more areas. Here, we present an all-optical implementation of a PGM through the sum-product message passing algorithm (SPMPA) governed by a wavelength multiplexing architecture. As a proof-of-concept, we demonstrate the use of optics to solve a two node graphical model governed by SPMPA and successfully map the message passing algorithm onto photonics operations. The essential mathematical functions required for this algorithm, including multiplication and division, are implemented using nonlinear optics in thin film materials. The multiplication and division are demonstrated through a logarithm-summation-exponentiation operation and a pump-probe saturation process, respectively. The fundamental bottlenecks for the scalability of the presented scheme are discussed as well.

## Abstract (web summary)

To circumvent the limitations of electronic computers, moving to hybrid optical-electronic or all-optical devices may be useful. Here, Babaeian et al. present an all-optical implementation of the probabilistic graphical model using nonlinear optics in thin films to implement mathematical functions.

## Introduction

One of the major challenges in electronic computation is the optimization problem that usually occurs in a large data set where each variable depends on or has influence on other variables. The PGM is a standard and extremely powerful approach to calculate the joint probability distribution for a large number of variables where each element of the set depends on other variables^1–5^. PGM methods are used in a variety of fields including social networks^6^, artificial intelligence^7, 8^, machine learning^8–11^, decision-making, speech recognition, image processing^12^, and computational biology^13–19^. Electronic central processing units are not the best tools to address these problems. Introducing multicore technology and parallel computing architectures such as sub-threshold very large-scale integration, application-specific integrated circuit (ASIC) and a custom ASIC, the Tensor Processing Unit from Google^20^, have improved speed/power cost for optimization problems, but optimization problems for big data remain a big challenge. Heat generation and bandwidth limitations of electronic devices are the main reasons for this, and reports of Moore’s law being exhausted have become common^21–23^. For these reasons, hybrid optical-electronic accelerators have recently been explored to improve electronic digital computing in terms of speed enhancement and energy efficiency for several problems such as signal processing^24–29^, spike processing^30–32^, and reservoir computing^33–35^.

**Fig. 1:**
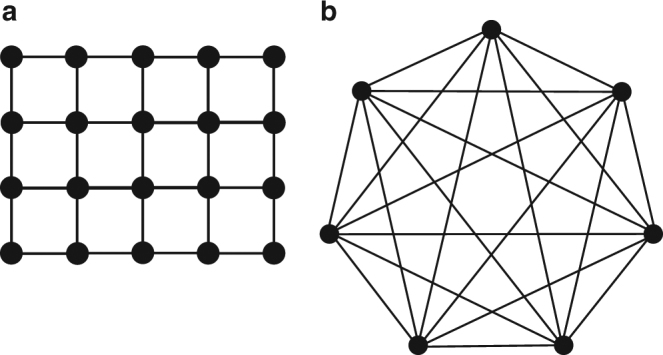
Graphical maps with different node connectivity. **a** Locally connected graph. **b** Fully connected graph

The SPMPA is commonly used in graphical models. In this algorithm, a message (µ_*S*→*R*_) containing the influence that node *S* exerts on node *R* is passed to *R*. When node *R* is connected to multiple nodes, the message received at *R* is the normalized product of the influences from all other nodes,

1 $$p\left( {x_1,x_2, \ldots x_n} \right) = \frac{1}{Z}\mathop {\prod }\limits_{n = 1}^N x_n$$

where *Z* is a normalization factor, *p* is the probability distribution and *N* is the total number of nodes. Graphically, each variable is represented by a node and its potential to be influenced by other nodes is represented by the connections to other nodes or edges^2^ (Supplementary Note 1). For instance, Fig. 1a shows a graph for image processing with each node representing a pixel in the image that is being influenced by its four nearest neighbors; thus 4 edges for each node with an alphabet *K*, defined by the potential intensity of each pixel, *K* = 256 for 8-bit encoding in each pixel. Figure 1b shows a fully connected graph that is applicable to, e.g., an Ising problem with each node representing an electron in a solid with its spin influenced by all other electrons with *K* = 2 for spins up or down.

A fully optical implementation of PGMs, using a wavelength multiplexing architecture could offer a promising approach to efficiently solving large data set problems, potentially providing benefits such as increased speed and lower power consumption (Supplementary Note 2). However, we must note that with current coherent laser technologies and known nonlinear optical materials in nature, there are some fundamental problems in order to scale the number of nodes to very large number (e.g., 10^6^). We discuss the fundamental challenges later in the Results section. In this paper, we present our experimental results on the optical implementation of the wavelength multiplexed architecture of message passing algorithm of PGMs for *N* = 2 and demonstration of the mathematical functions, including multiplication and division, using nonlinear optics.

## Results

### Wavelength multiplexing architecture

**Fig. 2:**
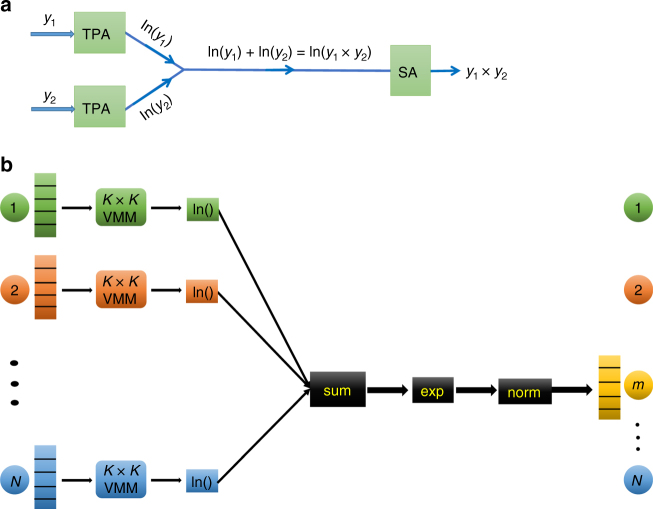
Wavelength multiplexing architecture. **a** ln-sum-exp scheme to multiply two numbers. **b** Schematic representation of the sum-product message passing algorithm (SPMPA) for node *m*. The spectral bandwidth is divided equally as a representation of each node (different color indicates different wavelength). The summation unit sums across wavelength for each probability vector that emerges from the natural logarithm (ln) modules

The multiplier of the message passing algorithm of Eq. (1) can be written with natural logarithmic (ln), summation and exponential operations (Fig. 2a) as,

2 $$\mathop {\prod }\limits_{m = 1}^j Y_m = {\mathrm{exp}}\left( {\mathop {\sum }\limits_{m = 1}^j {\mathrm{ln}}\left( {Y_m} \right)} \right)$$

This embodiment of the multiplier is easier to implement optically. In the wavelength multiplexing architecture of Fig. 2b, each node is represented by a different wavelength shown by a different color, since the spectral bandwidth can be equally divided and used for each node.

The graph in Fig. 2b has *N* nodes and the alphabet size is *K*. To find the updated probability vector of the target node (node *m* in Fig. 2b), each message from its neighbor nodes is first multiplied with a compatibility matrix whose elements are conditional probabilities^2^. This operation is called vector-matrix-multiplication (VMM). The outputs of the VMM are then multiplied element-wise and normalized to yield the updated probability vector of the target node. The product of all messages is replaced with logarithmic, summation and exponential operations as shown in Eq. (2). These operations are applied to every node in order to determine its updated probability vector. The updated vectors are then used in subsequent iterations until their values reach steady state. Thus, the two most important mathematical operations required to compute the probability vector are multiplication and division for normalization^36^. The natural logarithmic function can be implemented optically by two photon absorption^37^ (TPA), while the exponential function can be optically realized through saturable absorption (SA), and the summation function by the fan-in process, respectively. However, using analog optics to implement the mathematical functions and operations can induce noise^38–40^, which can affect the performance of the optical solution of the PGMs. Therefore, we performed simulations to determine the effect of noise on the failure rate of the sum-product message passing algorithm. Our results indicate a 99% success rate for a graph with one-million nodes, an alphabet size of 100 and 20% connection density. In other words, the optical implementation of the sum-product message passing algorithm is very tolerant to the noise (Supplementary Note 3).

### Multiplication

Inserting the saturable absorption equation $\alpha \left( I \right) = \alpha _0/\left( {1 + I/I_{{\mathrm{sat}}}} \right)$ in the differential equation for the nonlinear absorption^41^, ${\mathrm d}I/{\mathrm d}z = - \alpha \left( I \right)I$ and solving leads to

3 $$I_{{\mathrm{out}}}{\mathrm{e}}^{\frac{{I_{{\mathrm{out}}}}}{{I_{{\mathrm{sat}}}}}} = I_{\mathrm{in}}{\mathrm{e}}^{\frac{{I_{{\mathrm{in}}}}}{{I_{{\mathrm{sat}}}}} - \alpha _0L}$$

**Fig. 3:**
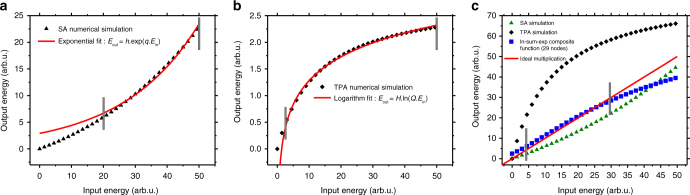
Numerical simulation of multiplication. **a** Comparison of the saturable absorption (SA) solution with an exponential function *E*_out_ = *h*.exp(*q*.*E*_in_). Fit coefficients are *h* = 2.906 and *q* = 0.041 and parameter values of the numerical simulation are *α*_0_ = 5(arb.u.),*E*_sat_ = 70(arb.u.) and *L* = 0.3(arb.u.). **b** Comparison of two photon absorption (TPA) solution with a natural logarithm function *E*_out_ = *H*.ln(*Q*.*E*_in_). Fit coefficients are *H* = 0.646 and *Q* = 0.723 and parameter values of the numerical simulation are *α*_0_ = 5(arb.u),*L* = 0.3(arb.u.) and *β* =0.5(arb.u.). **c** The blue squares show the composite mathematical operations of ln-sum-exp for 29 inputs and the solid red line represents ideal multiplication. The normalized-root-mean-square (NRMSE) of less than 1% (between simulated multiplication and the ideal multiplication) occurs between the bounded range, which is input energies between about 19 (arb.u.) to 32 (arb.u.)

Here *I*_sat_ is the saturation peak irradiance, *α*_0_ is the weak field absorption, *L* is the thickness of SA material and *I*_in_ and *I*_out_ are the input and output peak irradiance, respectively. A numerical solution of Eq. (3) and its fit with an exponential function are plotted in Fig. 3a. Including the TPA term in the nonlinear absorption differential equation^42^ d*I* / d*Z*= −*α*_0_*I* − *βI*^2^, leads to an explicit analytical solution,

4 $$I_{{\mathrm{out}}} = \frac{{I_{{\mathrm{in}}}{\mathrm{e}}^{ - \alpha _0L}}}{{1 + \beta L_{{\mathrm{eff}}}I_{{\mathrm{in}}}}}$$

where *β* is the TPA coefficient and $L_{{\mathrm{eff}}}\, = \,\left( {1\,-\,{\mathrm{e}}^{ - \alpha _0L}} \right)/\alpha _0$. A numerical solution of Eq. (4) is plotted in Fig. 3b as well as its fit with a natural logarithm function. The result of combinations for 29 identical logarithm inputs and an exponentiation gives the multiplication of the inputs as illustrated in Fig. 3c. The ideal multiplication result is plotted as a linear fit in Fig. 3c. Note that the peak irradiance in Eq. (3) and (4) can be replaced with energy per pulse (fluence or photon number as well) without any change in concept of their comparison with the exponential and logarithm functions. We use energy per pulse (*E*) for simulation as the experimental data were measured in terms of energy per pulse. In Fig. 3a and Fig. 3b we need to limit the range of fitting in order to get maximum overlap of the exponential and natural logarithm fit functions with SA and TPA solutions. Also the normalized-root-mean-square error (NRMSE) should be less than 1% and is defined as

5 $${\mathrm{NRMSE}} = \frac{{\sqrt { \langle \left( {E_{{\mathrm{out}}} - E_{{\mathrm{fit}}}} \right)^{^2}} \rangle }}{{E_{{\mathrm{max}}} - E_{{\mathrm{min}}}}}$$

Limiting the ranges also comes from the natural behavior of the SA and TPA process where Eq. (3) and (4) start from zero for no input energy. However, we know that e^0^ = 1 and ln(0) is undefined. Therefore, bounding the input intensity range for fitting is necessary for convergence and adequate fitting of the solutions of the TPA and SA equations with the target functions. The criteria are the maximum error acceptable to reproduce the function.

**Fig. 4:**
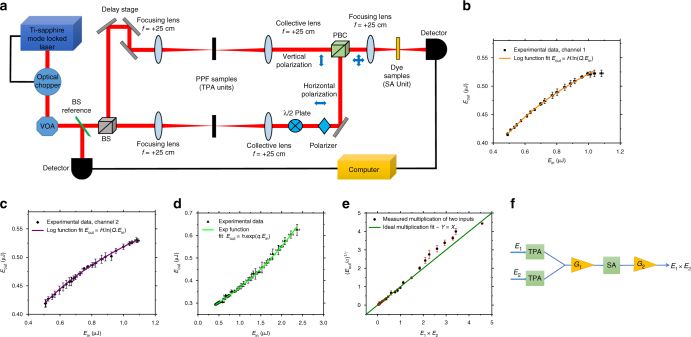
Experimental multiplication results. **a** Experimental setup to multiply to input energies. A variable optical attenuator (VOA) and a beam splitter (BS) are used to monitor the input energies to the two photon absorption (TPA) units. A polarization beam combiner (PBC) was used to combine the input energies from two arms as well as preserving their polarization in order to avoid interference at saturable absorber (SA). **b**, **c** Experimental TPA data (square) and the nonlinear fit *E*_out_ = *H*.ln(*Q*.*E*_in_) (solid lines) where *H* = 0.148 µJ and *Q* = 33.663 µJ^−1^. The error bars denote the standard deviation of reading input and output energy per pulse for 200 shots for each data point. **d** Experimental SA data (triangle) and the nonlinear fit *E*_out_ = *h*.exp(*q*.*E*_in_) (solid line), where *h* = 0.247 µJ and *q* = 0.401 µJ^−^. The error bars are the same as in **b**, **c**. **e** The measured final energy output vs. multiplication of the input values. The solid green line shows ideal multiplication. The error bars show the relative percent error between experimental readout and ideal multiplication of two energy inputs. **f** Modification of Fig. 2a schematic capable of performing an ideal multiplication. It requires two gain blocks, *G*_1_ and *G*_2_ in which the values of the gains depend on the material and the experimental setup

We have performed the multiplication experiment to multiply two energies in thin film materials. Fig. 4a denotes the experimental setup for the multiplication experiment. The thickness of the TPA and SA devices are 50 ± 2 nm and 3 µm, respectively. The material that was used to produce the natural logarithm function in the TPA units was amorphous carbon made by the pyrolyzing photoresist film (PPF) technique^43, 44^ (Supplementary Note 5) and a nonlinear optical dye (thiopyrylium-terminated heptamethine cyanine) as the SA^45^ to achieve the exponential function (Supplementary Note 6). Fig. 4b, c, d show *E*_out_ vs. *E*_in_ and the nonlinear fit functions with the natural logarithm and exponential functions for the TPA blocks and SA block, respectively. As we expected, according to the TPA and SA simulations, the logarithm and exponential function fits do not have exact mathematical form of ln(*x*) and e^*x*^ due to the weak field, TPA, scattering, and the insertion loss from optical components. However, the fit coefficients (*H*, *Q*, *h*, *q*) are known and constant, so that we can take these coefficients into account as imperfections that cause deviations from the exact mathematical multiplication. Considering the Maclaurin expansion of Eq. (3) and the fit function in Fig. 4d up to third order, we define the coefficient *q* to be proportional to $\alpha _0L/I_{{\mathrm{sat}}}A_{{\mathrm{eff}}}$, where *A*_eff_ is the spot size of the optical beam. On the other hand, as we explained in Fig. 2a, the composite function of the sum of two natural logarithm functions and subsequent exponentiation yields the product of input values. Now taking the fit coefficients from Fig. 4b and Fig. 4c in account, we get the summation of the two output values from the TPA blocks as:

6 $$H.{\mathrm{ln}}\left( {Q.E_1} \right) + H.{\mathrm{ln}}\left( {Q.E_2} \right) = {\mathrm{ln}}\left[ {\left( {Q^2.E_1 \times E_2} \right)^H} \right]$$

Here the polarization beam combiner (PBC) does the summation operation and this is the value out of the PBC and the input to the SA. The SA operates on the input values based on the fit equation in Fig. 4d:

7 $$h.{\mathrm{exp}}\left[ {q.{\mathrm{ln}}\left( {\left( {Q^2.E_1 \times E_2} \right)^H} \right)} \right] \to h\left( {Q^{2H.q}} \right)\left( {E_1 \times E_2} \right)^{H.q}$$

Eq. (7) reduces to *σ*(*E*_1_×*E*_2_)^*γ*^, where *σ* = *hQ*^2*Hq*^ and *γ* = *Hq*. The numerical values for the experimental setup and materials that we used are *σ* = 0.375 and *γ* = 0.059. These coefficients capture all of the imperfections and fundamental material characteristics of the setup. However, in order to get pure mathematical multiplication of two numbers as desired, we can add two gain blocks in the setup to eliminate the *σ* and *γ* coefficients and get exactly *E*_1_ × *E*_2_. Fig. 4f shows a schematic of these modification where *G*_1_ and *G*_2_ must be equal to 1/*γ* and 1/(*hQ*^2^), respectively. Note that, based on conservation of energy, fundamentally we cannot take two energy values and detect their direct multiplication. Hence, adding gain blocks is quite reasonable although this increases the power consumption of the computation. However, If we want to multiply more than two numbers in which *σ* or *γ* or both become greater than one, we need to add attenuation blocks instead of *G*_1_ or *G*_2_. The selection of gain block(s) or attenuation block(s) depends on the size of the graph, number of edges, and the material characteristics. Fig. 4e shows the measured output energy as a result of appropriate manipulations of the two inputs, vs. the desired multiplication of the two numbers. We have included the optical constants *σ* and *γ* in the output values to demonstrate that the simulation matches with the experiment. As can be seen, the range of *E*_1_ × *E*_2_ values between 0 and 1.3 has a minimum error of less than 1%, as we expect to observe. Based on Fig. 4b, c, the dynamic range for which the TPA blocks provide the natural logarithm function is between 0.5 µJ to 1.1 μJ (3.5 dB). Therefore, multiplication of these numbers results in a maximum of 1.21. For numbers outside of the dynamic range of the TPA and SA units, the output values exhibit greater deviation from the desired multiplication values as can be seen from comparison with the solid green line in Fig. 4e.

### Normalization

**Fig. 5:**
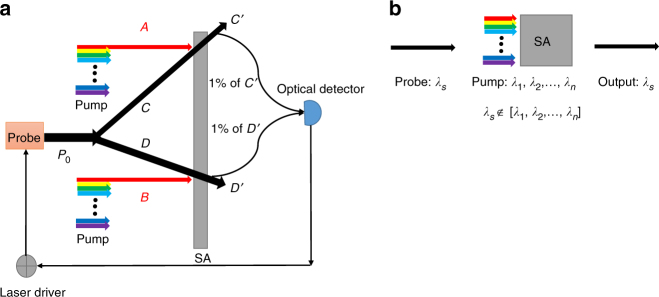
Normalization and wavelength remapping. **a** Schematic setup to normalize two numbers using a pump-probe saturation experiment. **b** Wavelength remapping concept where each element of the probability vector is modulated in the presence of a broadband pump, requiring spatial separation in the saturable absorber (SA)

According to Eq. (1) the normalization factor (*Z*) must be taken into account to ensure that the probability vector distribution is mapped between zero and one. For normalizing the probabilities that we get from the multiplication of each node, we decided to use an optical pump-probe saturation setup followed by an electrical feedback-loop system. For this operation, we employ a SA such that by increasing or decreasing the pump intensity, approaching saturation, we can increase or decrease the optical intensity of the probe beam. The concept for normalization of two power inputs *A* and *B* is described in Fig. 5a. The SA is used to (1) make the sum of all elements of each normalized probability vector constant and (2) integrate over the input spectrum and translate to proper node-specific output wavelength. In the feedback-loop, the adjustable power *P*_0_ is such that for any value of *A* and *B*, *C*′ + *D*′ remains constant, where *C*′ = *P*_0_*A/*(*A* + *B*) and *D*′ = *P*_0_*B*/(*A* + *B*). According to the message passing algorithm, implemented via a wavelength multiplexing approach, the information in the probability vector should be recirculated for the next iteration and they must be monochromatic. However, the receipt node receives multiple wavelengths from the pump. The pump is a broadband coherent source that enforces the value of the probability vectors and the probe is a constant signal at the node’s wavelength. The output power is modulated with pump intensity and has the same wavelength as the probe. We should also note that the individual elements of the probability vector must be spatially separated in the SA. Thus the element will be modulated separately in the presence of pump intensity. Fig. 5b shows a schematic of the wavelength remapping through the pump-probe saturation process.

**Fig. 6:**
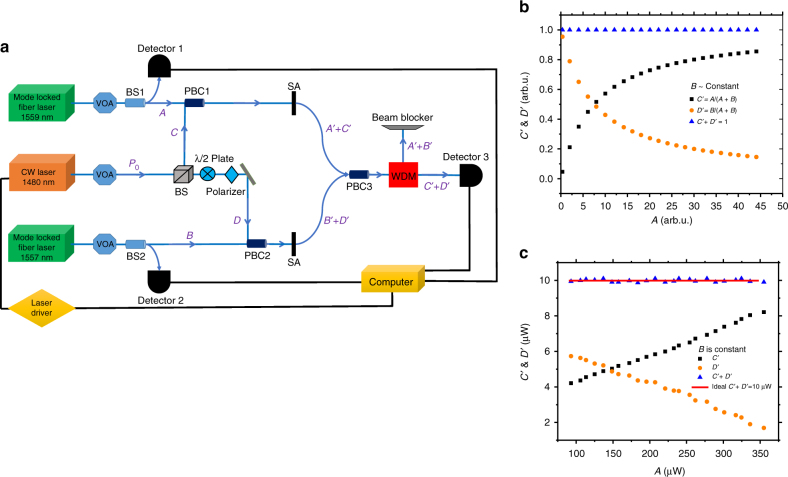
Simulation and experimental results of normalization. **a** Experimental setup to normalize two powers *A* and *B*. The pump sources are two mode-locked fiber lasers. The characteristics of these lasers are as follows: *λ*_A_ = 1559 nm with 8 MHz repetition rate and 200 fs pulse width, and the other one *λ*_B_ = 1557 nm, 109 MHz, and 240 fs pulse width. The probe was a continuous wave (CW) diode laser *λ*_probe_ = 1480 nm. Three variable optical attenuators (VOAs) and two beam splitters (BS1 and BS2) were used to monitor input powers to saturable absorbers (SAs). The polarization beam combiners (PBCs) were also used to combine the pump lasers powers with probe laser power with preserving their polarization. And a wavelength-division multiplexing (WDM) device was used in order to separate the modulated probe laser from the modulated pump lasers (see Methods for detail). **b** Simulated result to normalize two numbers *A* and *B* where we assume *B* is constant. **c** Experimental result to normalize two powers. In both **b**, **c**, the feedback-loop system adjusts the modulated power of *C*′ + *D*′ to remain constant

Fig. 6a denotes the experimental setup for normalizing two powers where we used chemical vapor deposition (CVD) grown graphitic pyro-carbon (GrPyC)^46, 47^ thin films that were transferred onto two fiber tips as the SAs. The thickness of the samples was 50 ± 2 nm. (Supplementary Note 4). Figure 6b shows the simulation of an ideal normalization of two input powers *A* and *B* and the result of *C*′ + *D*′ = 1 (arb.u.). Here we assume that the optical power *B* is constant and the feedback-loop mechanism is employed to control *P*_0_ such *C*′ + *D*′ remains constant. Fig. 6c shows the experimental result and demonstrates good agreement with the simulation. In the experiment we kept *B* at a constant value of 100 µW and set the output of the CW laser after SAs to be 10 µW, which is the desired constant value that we want to achieve in presence of laser powers *A* and *B*. It has been shown that increasing the intensity of laser *A*, increases the output of the probe laser at the corresponding arm, *C*′, and accordingly, the output in the other arm, *D*′, decreases because of the feedback-loop that keeps the *C*′ + *D*′ to be almost constant. The NRMSE of the experimental result vs. the ideal normalization in Fig. 6c (Red solid line) is about 1%.

## Discussion

One of the major challenges in the wavelength multiplexing architecture to solve PGMs is the scalability for a very large number of nodes (e.g., 10^6^). Hypothetically, increasing the spectral bandwidth of the coherent laser sources can result in an increase of the number of nodes. However, considering the current coherent source technologies, dividing the spectral bandwidth of the coherent source to a very large number, in order to represent each node, reduces the peak irradiance by several order of magnitudes. This reduction of the peak irradiance does not leave enough fluence to access the nonlinear TPA and SA behaviors of most known nonlinear optical materials in nature (Supplementary Note 7). Although, materials engineering may provide a route towards tuning the atomic line-shape, so that the lifetime can be longer. Coupling this with the tuning of the input frequency to that of one and two photon excited states, can enhance the cross section of TPA and SA processes such that a lower peak irradiance TPA and SA can be achieved.

We investigated, both theoretically and experimentally, the essential required mathematical functions to optically implement the message passing algorithm for probabilistic graphical models. The two basic and central mathematical operations, multiplication (through natural logarithm-sum-exponent operation), and division (normalization), which are required for the SPMPA, are optically implemented. Nonlinear thin film optical materials were employed for TPA (PPF^43, 44^) and SA (thiopyrylium-terminated heptamethine cyanine^45^) to demonstrate optical implementation of natural logarithm and exponentiation functions, respectively. We also used another type of nonlinear thin film as a saturable absorber (GrPyC^46, 47^) to implement normalization through a pump-probe-saturation experiment. Furthermore, with respect to the enormous breadth of applications that these two fundamental mathematical operations (multiplication and division) provide, the presented techniques can be used widely to enable these operations where they are used heavily. To estimate the speed of computation of the proposed optical PGM machine, we note that the multi photon excitation processes in the SA and TPA components, are extremely fast, in the sub-femtosecond range. So the rates of generating and detection of the light are the main time constraint of the overall system. For pulsed lasers the repetition rates can be larger than 100 Gbps^48^, while photodetectors can be as fast as 100 GHz^49^ as well. It should be pointed out that one of the advantages of the optical analog computation is that the speed of calculation will not increase as the problem increases in scale. Contrary to their analogous electrical devices, all the mathematical units presented here (ln, sum, exp, and norm) use optical components that do not require an external source of energy to perform the operation on the signal. In principle, using such passive elements could be a great benefit in terms of energy consumption^21^. However, optical insertion loss, as well as linear and nonlinear absorptions should be included into the energy budget, especially when the signal (which carries the energy) needs to be recirculated and when performing cascading operations^50^. For this reason, buffering amplifiers are required for optical implementation of the SPMPA approach for the PGMs. As a proof-of-concept an optical implementation of the PGM message passing algorithm for a two node graph (*N* = 2) has been shown successfully. A large-scale system-level demonstration for a larger number of nodes with high connectivity is the subject of ongoing work.

## Methods

### Data analysis

All numerical simulations for multiplication and normalization are done with MATLAB R2016a (MathWorks) and FORTRAN 90.

### Multiplication experiment

The optical laser source that has been used for this experiment was an 810 nm Ti-Sapphire laser, producing 150 fs pulse width (at FWHM) and a 50 Hz repetition rate. The original repetition rate out of the amplifier locked to the laser was 1 kHz, and using an optical chopper, synchronized and externally triggered with the amplifier pulses’ phase, allowed us to reduce the repetition rate to 50 Hz in order to reduce the probability of heat damage and thermal effects in the samples. Figure 4a is the schematic of the experimental setup, where in the TPA portion two convex lenses are used to increase the intensity and access the nonlinear absorption behavior of the samples, while the other two convex lenses are used for collecting and re-collimating the beam. The spot size at the focus was 76 µm. A half-wave plate and polarizer are placed in the path of one arm to insure that the output polarization result is perpendicular to the other arm’s polarization and a polarization beam combiner (PBC), which preserves the inputs polarizations orientation, combines the two beams with a perpendicular polarization orientation. Therefore, these two beams do not interfere at the SA even though they have same wavelength. Furthermore, a delay stage is installed for pulse synchronization, followed by an auto-correlator at the SA with femtosecond resolution. A variable optical attenuator and a beam splitter (BS) are used to monitor the input energies to the TPA units.

### Normalization experiment

Two femtosecond mode-locked fiber lasers were used as the pump sources, together with a CW laser probe. We have also used a half-wavelength plate and a polarizer in one of the probe laser’s path to avoid interference at detector number 3. BS1 and BS2 are used for power monitoring of *A* and *B* values. PBC1 and PBC2 combine power *A* with *C* and power *B* with *D* and make them collinear at the SAs, where the powers of *C* and *D* are modulated in the presence of pump lasers *A* and *B*, respectively. PBC3 combines all powers and a wavelength-division multiplexing (WDM) separates the two wavelengths since the wavelengths of lasers *A* and *B* are so close. An electronic feedback-loop system is used to control the probe laser power such that *C*′ + *D*′ remains constant for arbitrary numbers for *A* and *B*. However, this system has a finite dynamic range where *C* and *D* can be modulated in presence of *A* and *B* due to the weak field and nonlinear absorption range of SAs, as well as the damage thresholds of the samples. A LabVIEW-based code (National Instruments) was used for the feedback-loop system and adjusted the power output of the probe laser based on the reading from the three power meters.

### Data availability

The data that support the plots within this paper and other findings of this study are available from the corresponding author on reasonable request.

## Electronic supplementary material

Supplementary Information
