# Supplementary material for: Nonlinear optical components for all-optical probabilistic graphical model
Source: Nat Commun. 2018 May 29;9:2128. doi: 10.1038/s41467-018-04578-x (PMC5974078; doi:10.1038/s41467-018-04578-x)
Supplement: Supplementary file 1 — Supplementary Information [file 41467_2018_4578_MOESM1_ESM.pdf]

Supplementary Information

Nonlinear Optical Components for All-Optical Probabilistic Graphical Model

Masoud Babaeian[1,2 *], Pierre-A. Blanche[2], Robert A. Norwood[2], Tommi Kaplas[3], Patrick Keiffer[2], Yuri Svirko[3], Taylor G. Allen[4], Vincent W. Chen[4], San-Hui Chi[4], Joseph W. Perry[4], Seth R. Marder[4], Mark A. Neifeld[2, 5] and Nasser Peyghambarian[2]

[1]*Department of Physics, University of Arizona, Tucson, AZ 85721, USA*

[2]*College of Optical Sciences, University of Arizona, Tucson, AZ 85721, USA*

[3] *Institute of Photonics, University of Eastern Finland, Joensuu, FI 80101, Finland*

[4]*School of Chemistry and Biochemistry, Georgia Institute of Technology, Atlanta, GA 30332, USA*

[5]*Electrical and Computer Engineering, University of Arizona, Tucson, AZ 85721, USA*

* Corresponding author: Babaeian@physics.arizona.edu

Supplementary Note 1: Sum-Product Message Passing Algorithm

Sum-product message passing algorithm (SPMPA) is a standard and effective method to compute the marginal distribution for unobserved variables and to recover information based on the incomplete or noisy observations[1]. Supplementary Figure 1 denotes the SPMPA for the node i which is assumed to connect to j neighboring nodes. Each node transfers its message via multiplication of its probability vector with the compatibility matrix as $Y_i = C_{ij}\mathbf{V}_j(t)$ where C_{ij} is a compatibility matrix between node i (receiver node) and j (sender node) and $\mathbf{V}_j(t)$ is the probability vector of node j at time t. This operation called vector-matrix-multiplication (VMM). Then the messages from all neighbors are multiplied together as $W_i(t) = \prod_{m=1\neq i}^{j} Y_m(t)$ which this product needs to be normalized to ensure sum of the probability vector elements is 1 ($\mathbf{V}_i(t+1) = \mathrm{Norm}(W_i(t))$). This vector is the updated probability vector of node i. These operations iteratively apply to every node until the updated probability vector values reach at the steady state. The final probability vector is then considered to decide the state of the node.

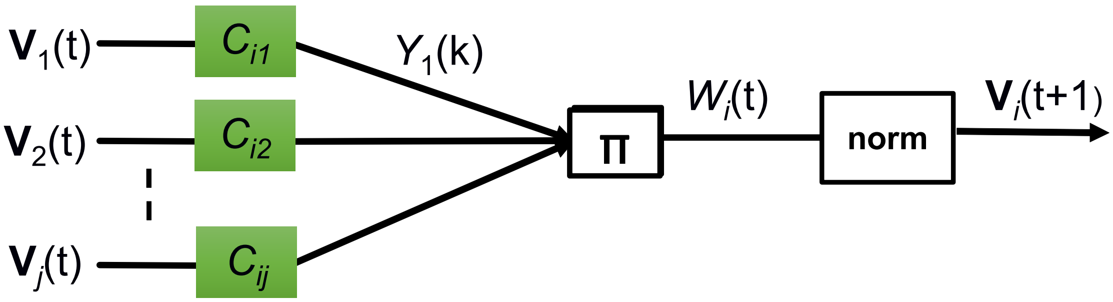

Supplementary Figure 1. Sum-product message passing algorithm for node *i*. Node i is connected to j neighboring nodes where j=1, 2, …, N. C_{ij} is the compatibility matrix between node I and j and $\mathbf{V}_j(t)$ is the probability vector of node j. Y_i and $W_i(t)$ are defined as $Y_i = C_{ij}\mathbf{V}_j(t)$ and $W_i(t) = \prod_{m=1\neq i}^{j} Y_m(t)$ respectively.

Supplementary Note 2: Wavelength Multiplexing Architecture

In order to minimize power consumption and the number of components for the SPMPA, we studied different multiplexing strategies. The most promising approach is to encode each node with different wavelength. The spectral bandwidth can be divided to several number of channels using arrayed-waveguide grating (AWG) technique[2-4]. This wavelength multiplexing indicates that the subsequent optical operations must be broadband, as must the coherent source, in order to get large of number of nodes (for instance, a broadband frequency comb source[5-10]). We should mention also the subsequent nodes must have enough peak irradiance to access the saturable absorption (SA) and two photon absorption (TPA) behavior of the nonlinear optical materials.

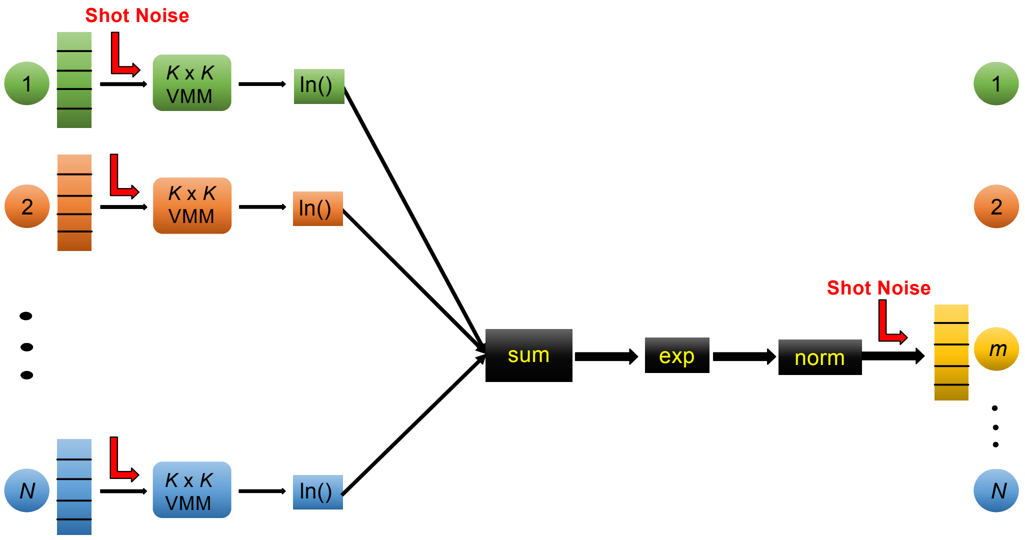

Fig. 1c

Supplementary Figure 2. Wavelength multiplexing approach for the sum-product message passing algorithm. The natural logarithm (ln), summation (sum) and exponentiation (exp) units has been substituted with product unit at Supplementary Figure 1 since they can be realized optically and can implement basic multiplication operation. Each color represents different wavelength for each node as the strategy for the wavelength multiplexing treatment. The vector-matrix multiplier (VMM) units are in a $K \times K$ dimensions where K is the alphabet size.

Supplementary Figure 2 shows the SPMPA, N nodes and the alphabet size of K, in the wavelength multiplexing layout where the spectral bandwidth is equally divided and used as a representation of each node in the graph. Each node also has a probability vector ($\mathbf{V}_j(t)$) of size K where the elements of $\mathbf{V}_j(t)$ vector denote the node has alphabet 0, 1, 2, …., K-1 respectively. Each VMM has dimension of $K \times K$ which requires K^2 spatial resources each with N wavelength channels. Therefore, for an N node graph and an alphabet size of K, the number of VMM modules will be $K^2 \times N \times D$ where D is the connection density in the graph. Accordingly, the number of modules for natural logarithm (ln), summation, exponential and normalization will be $K \times N \times D$, $K \times N$, $K \times N$ and $K \times N$ respectively. Hence the implementation of the graph with N nodes and an alphabet size of K needs $K^2 \times N \times D + K \times N \times D + 3K \times N$ components. For a fully connected graph with size of N equal to 10^6 nodes and $K = 100$, the system requires roughly 10^{10} components. To estimate a lower bound on power requirement to implement such graph with N equal to 10^6 nodes, $K = 100$ and 20% connection density, we considered 10^4 photons as the shot noise detection limit and 1 μm wavelength and 1 ms iteration time. The total energy requirement for 100 iterations to get the final answer is roughly 200 J. Assuming 1ms /iteration, this would lead to a 200 kW power requirement. We should mention this is a minimum power requirement and it excludes loss in the optical components, power needed for light source and its modulation, etc. However, the lower bound electronic power required to solve such that graph (digital CPU platform) is orders of magnitudes higher than the optical solution (roughly ~1.5 GW with an Intel Knight's Landing architecture with 72 cores at 6 TFLOPS for computation[11] and PCIe and 100 GB Ethernet for the communication). We also estimate the power consumption to solve such problem with other electronic platforms such as application-specific integrated circuit (ASIC) and sub-threshold very large scale integration (VLSI). We have

determined 1.4 GW and 1.7 MW for ASIC and VLSI platforms respectively. Additionally, a custom type of ASIC, called a Tensor Processing Unit (TPU) by Google[12], have proved to be nearly 200 times (speed/power) better than CPU platforms for neural network problems. However, this is still far from an ideal and efficient machine for optimization of a large number of nodes in a graph model problem. We note that our assumptions are aggressive and disregard some peripheral components that would also need power. For instance, the power computation for the electronic platforms excludes power consumption of the electronic drivers. Nevertheless, these values are lower bounds, and the comparison points to a substantial potential advantage for the optical solution. The key advantage of optical implementation is the optical components are passive which by passive we mean there is no need to apply external power to do the mathematical operations[13] (ln, sum, exp and normalization). The SPMPA implementation via wavelength multiplexing also is not bounded with enormous power consumption for communication unlike the electronic platforms which the power for communication is highly dominate compare to the power for computation[14-16].

Supplementary Note 3: Effect of Shot Noise on The SPMPA Performance

We performed a theoretical simulation to investigate the effect of noise on the performance and robustness of the optical implementation of the SPMPA. The modeling indicates a new lower bound on power consumption. Supplementary Figure 2 also denotes where the shot noise is added to the algorithm. Shot noise is inserted for each node before VMM operation where the photons are generated. After normalization units we also added shot noise to make sure that each

node starts with same number of photons for the next iteration. To study about effect of shot noise, photon number is used in the modeling for VMM, natural logarithm, exponential and normalization operations. The shot noise expression is inserted as $n_i = n_{0i} + \mathrm{Gauss}(0, \sqrt{n_{0i}})$ where n_{0i} is the initial photon number injected to each node, i is the node number and $\mathrm{Gauss}(0, \sqrt{n_{0i}})$ is the Gaussian distribution with standard deviation of $\sqrt{n_{0i}}$ and zero mean value. The numerical simulation was done with a Monte Carlo method where we first initiate a desired configuration. Then this configuration was used for the probability vector whose elements are all equal to 1/*K* except one node that we assume is known (e.g. a graph with *K*=3 in which probability vector would be [0.33, 0.33, 0.33]). The probability vector of the known node has one element which has higher value than rest of the elements that is corresponded to its assigned alphabet (e.g. a graph with *K*=3 in which probability vector for the known node could be [0.5, 0.25, 0.25]). After several iterations (when the steady state reached and the elements of final probability vector for each node got stabilized) simulation converges to its probable alphabet for each node. The result is a failure or a success if the simulated configuration is different or match with the desired configuration respectively. Our analysis indicates 99% success rate to optically implement the SPMPA for a graph with one million nodes, an alphabet size of 100 and 20% connections at the shot noise limit. In this regard we conclude that optical implementation of SPMPA through wavelength multiplexing is highly tolerant and robust to shot noise and imperfections.

Supplementary Note 4: Material Synthesis for SA Need for Normalization Experiment.

Graphitic pyro-carbon (GrPyC) was grown on a copper foil by hot wall chemical vapor deposition (CVD). The procedure was similar to conventional graphene synthesis on a copper foil but the amount of methane was increased in order to obtain a bit thicker graphitic film[17,18]. Copper foil (99,8 % pure) was first heated to 1000°C temperature in hydrogen atmosphere (0.5 mBar/5 sccm flow) and then methane was injected into the chamber. The process was done in static atmosphere, i.e. there was no gas flow in the chamber during graphitization. The pressure of the chamber was 23 mBar and the graphitization process lasted for 30 minutes. After graphitization the chamber was pumped down to vacuum and methane was replaced by hydrogen (10 mBar). The sample was cooled down overnight in a hydrogen atmosphere. The GrPyC film was next transferred from the copper foil on a fiber tip and on a silica substrate. The GrPyC film, transferred on a silica substrate was coated with a 500 nm thick poly (methyl methacrylate) (PMMA) layer, while the sample transferred on a fiber tip was transferred without PMMA. Backside carbon from the copper foil was cleaned with a short oxygen plasma (100 W / 1 min / 20 sccm). The copper foil was etched by the $FeCl_3$ solution and the graphitic film was cleaned with purified water[18]. Supplementary Figure 3 shows the characteristics of the GrPyC sample on a silica substrate. Low and high resolution scanning electron microscope (SEM) images show that the sample is rather uniform with some wrinkles similar to graphene. Transmission of the GrPyC film is almost constant ~90 %. Since absorption of a graphene monolayer is 2.3 % at all wavelengths, this result indicates a few layer graphene. The Raman spectrum of the GrPyC film shows a strong D peak (1350 cm^{-1}) and a widened G peak (1585 cm^{-1}). This indicates a rather high density of defects and amorphous carbon in the film[19,20]. Despite the highly defect density, the 2D peak can be observed at 2700 cm^{-1}. The 2D peak is not typical for fully amorphous

carbon (see e.g. Supplementary Figure 3e), but D and G peaks indicate strong disordering, so one can conclude that the material is nano-crystalline, a few atom layer thick graphite[20].

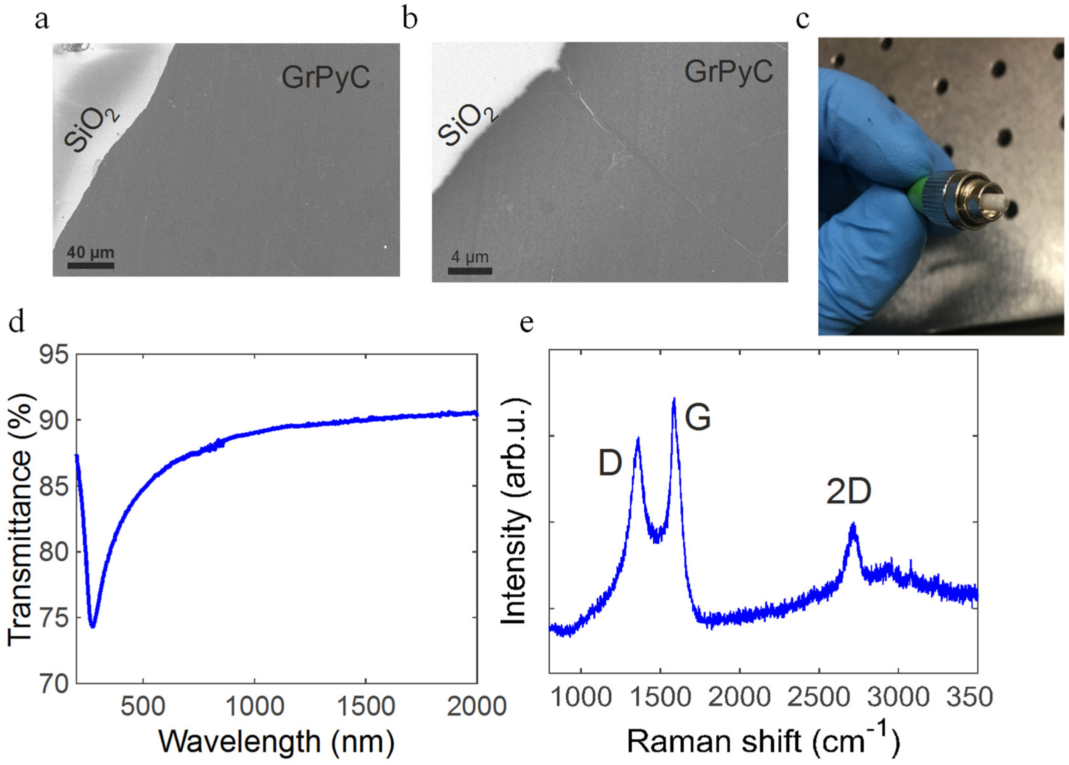

Supplementary Figure 3. Characteristics of the GrPyC sample. (**a**) Low magnification SEM image from a scratch show that GrPyC is rather uniform but due to the transfer from a copper to a silica substrate, some wrinkles appear. (**b**) High magnification scanning electron microscope (SEM) image of the sample. (**c**) A photo of transferred GrPyC sample onto a fiber tip. (**d**) Transmittance of GrPyC is almost constant at near infrared. Absorption peak at 260 nm resembles that of M-saddle point absorption of graphene. (**e**) Raman spectrum (averaged over five different points) shows strong D and G peaks but also the 2D peak is observable.

Supplementary Note 5: Material Synthesis for Two-Photon-Absorption Blocks in Multiplication Experiment

Amorphous carbon films can be fabricated e.g. by pyrolyzing photoresist in an inert atmosphere at rather high (~800 – 1100 °C) temperatures so-called pyrolyzed photoresist films (PPF) are amorphous carbon films[21,22]. The sample for the experiment was fabricated by spin coating about a 500 nm thick nLOF AZ-2070 resist layer on a 0.5 mm thick silica substrate. The sample was next heated with CVD to 800 °C in a hydrogen atmosphere (0.5 mBar/5 sccm flow).

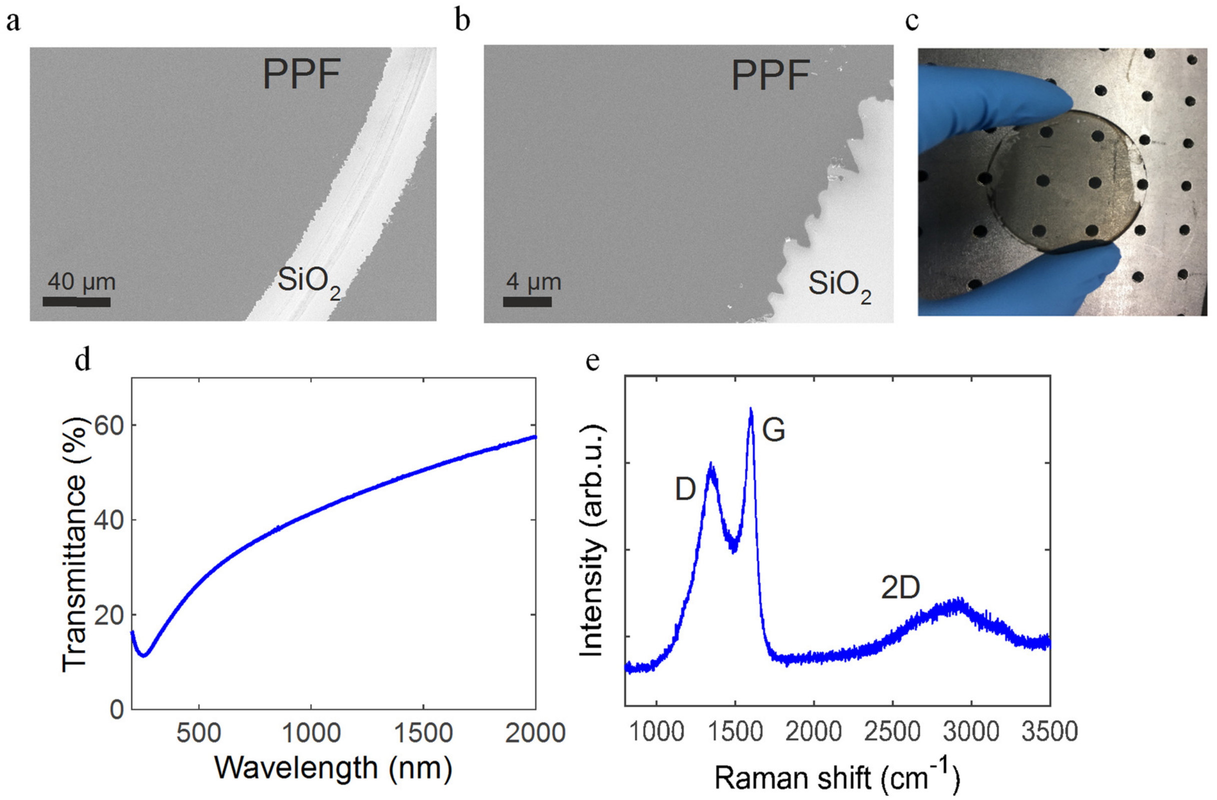

Supplementary Figure 4. Characteristics of the PPF sample. (**a**) Low magnification SEM image of pyrolyzed photoresist film. (**b**) High magnification scanning electron microscope (SEM) images of pyrolyzed photoresist film. The film is fully uniform over the substrate. (**c**) A picture of the PPF sample. (**d**) Transmittance of the PPF is not constant like in GrPyC but the absorption peak maximum is at 260 nm. (**e**) Widened D and G modes and absence of 2D peak in the Raman spectrum indicate highly amorphous carbon material.

800 °C temperature lasted for 5 minutes and the sample was then cooled down to room temperature (overnight) in a static, 5 mBar hydrogen atmosphere. Samples were characterized by scanning electron microscopy (SEM LEO 1550 Gemini), Raman spectroscopy (Renishaw Raman inVia Microscope) by using 514 nm excitation wavelength, and transmission spectroscopy (Perkin Elmer Lambda-9). The thickness of the PPF was measured by a stylus profilometer Weeko Dektak-150 and it was 50 ± 2 nm. Supplementary Figure 4 shows the properties of the PPF. By SEM the film appears very uniform throughout the substrate. The film thickness after pyrolysis was 50 ± 2 nm and transmittance about 50 % at 1500 nm. The transmittance was not as constant as it was for GrPyC. A strong D peak, widened D and G peaks and absence of 2D peak are all indicators that the carbon film is very amorphous but with dominating $sp^{(2)}$ hybridization[20,23].

Supplementary Note 6: Material Synthesis for SA Need for Multiplication Experiment.

The thiopyrylium-terminated heptamethine cyanine saturable absorber (structure in Supplementary Figure 5) used in this work was made as previously reported by Marder and Perry *et al*[24].

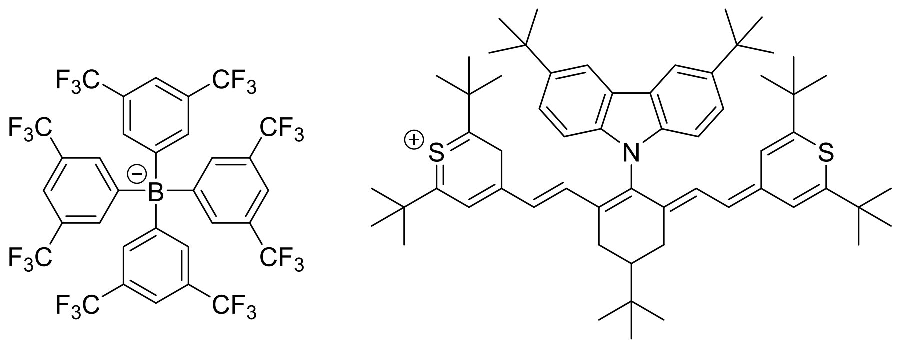

Supplementary Figure 5. Chemical structure of Thiopyrylium-terminated heptamethine cyanine saturable absorber.

Substrate Cleaning. Fused silica substrates were baked at 600 ^oC for 2 hours to burn off any organic residue. Substrates were then completely submerged in piranha (3:1 v/v H_2SO_4:30%H_2O_2) for one hour. Piranha-cleaned substrates were rinsed with copious amounts of water and then received a final rinse with methanol before being dried with a stream of nitrogen gas. Prior to spin coating, substrates were sonicated in acetone for 10 minutes and then dried with a stream of nitrogen before a second sonication step in isopropyl alcohol (IPA) for 10 minutes. The IPA sonication step was repeated two more times. To have the cleanest possible surface, nitrogen-dried substrates were immediately transferred to the spin coater after the final sonication step.

Polymer Purification. Poly (methyl methacrylate) (PMMA) (Sigma-Aldrich®, analytical standard for GPC, average MW ~97 kDA) powder was dissolved in 1,1,2-trichloroethane (TCE) to make a 12% w/w solution at 75 ^oC. The warm solution was then slowly poured into cold methanol while stirring, which yielded white clumps of precipitated PMMA. The precipitate was then vacuum filtered to dryness. The process of dissolving PMMA in TCE, precipitating in cold methanol, and drying under vacuum suction was repeated twice more. After three cycles, PMMA was dried under vacuum in an oven overnight at 100 ^oC.

50% w/w PMMA / Saturable Absorber Blend Films. To ensure the best quality measurements, it was critical to obtain high optical-quality blend films containing a high loading percentage of the saturable absorber. PMMA is a suitable polymer host for such films as it has been shown to dissolve a variety of conjugated organic dyes and produces high optical-quality spin coated films from a multitude of solvents[25-27]. 50% w/w PMMA / saturable absorber blend solutions were made by preparing 8% w/w solutions of purified PMMA and saturable absorber in spectroscopic grade dibromomethane (DBM) (Sigma-Aldrich® 99%) separately and then

combining equal masses of each. Before combining, the PMMA solution was stirred vigorously while heating at 50 ^{o}C and the saturable absorber solution was stirred vigorously in the dark with no heating to ensure complete dissolution of each component. After 30 minutes of stirring, the blend solutions were filtered using a 13 mm VWR® Nonsterile 0.2 µm PTFE Membrane Syringe Filter with Polypropylene Housing directly onto the substrates mounted in the spin coater, taking care to evenly cover the substrate. Prior to spin coating, the atmosphere within the spinning chamber was saturated with DBM vapor. Films were spun by accelerating at 100 RPM/s for 5 s to evenly spread solution over the substrate surface and then accelerating at 250 RPM/s to a top speed of 750 RPM, which was then held for 5 minutes. After spinning, films were quickly transferred to a desiccator and dried under vacuum overnight, protected from any light exposure (Supplementary Figure 6).

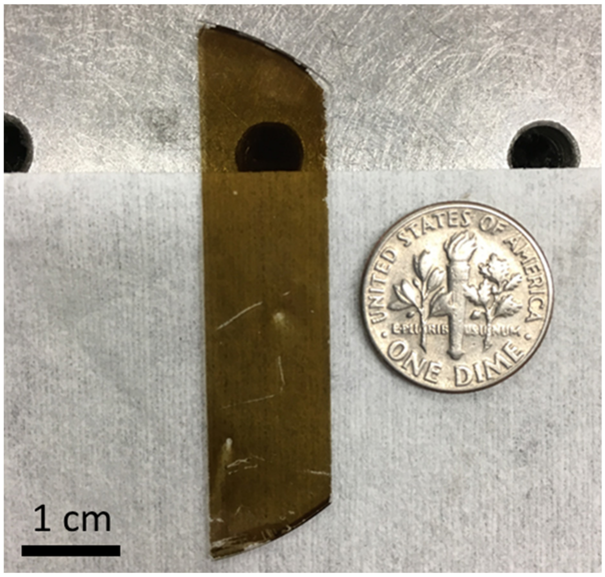

Supplementary Figure 6. Image of two substrates coated with 50% w/w PMMA / saturable absorber with thickness of 3 µm.

Supplementary Note 7: Scalability

In this section we estimate a rough number of nodes for a possible optical implementation of probabilistic graphical model (PGM) based on a wavelength multiplexing approach with a real laser system. We also discuss fundamental conditions of scalability for a large number of nodes based on current coherent laser source technologies and limitations of nonlinear optical material behavior. We consider laser pulses (wavelength λ=800 nm, spectral bandwidth $\Delta\lambda$=30 nm, pulse width $\Delta\tau$=50 fs, repetition rate=1 kHz, energy per pulse $E_{\text{per pulse}}$=1mJ) produced by a mode-locked Ti:Sapphire laser, which seeds a Ti:Sapphire regenerative amplifier. We search for maximum number of nodes that we can have in order for each node to have enough peak irradiance to enable TPA and SA in standard nonlinear optical materials. We have used the Fourier transform limit for a Gaussian beam profile, $\Delta\nu.\Delta\tau \geq 0.441$, to estimate the pulse width of each individual channel after dividing the spectral bandwidth into *N* channels:

$$\Delta\nu.\Delta\tau \geq 0.441 \tag{1}$$

Where $\Delta\nu$ is the frequency bandwidth measured at full-width at half maximum (FWHM) and $\Delta\tau$ is the pulse width at FWHM in time. Supplementary Equation 1 can be written in terms of spectral bandwidth and taking the divided spectral bandwidth domain to *N* channels in account, results:

$$\Delta\tau' \geq (0.441 \times \lambda^2 \times N)/(c \times \Delta\lambda) \tag{2}$$

Where *c* is the speed of light and $\Delta\tau'$ is the pulse width at FWHM in time for each individual channel. To calculate peak irradiance for each channel, we need to take the energy per pulse divided by *N* as well:

$$I_{\text{peak}} = 2E_{\text{per pulse}}/(\pi N \Delta\tau' r^2) \tag{3}$$

Where I_{peak} is the peak irradiance and r is the beam radius. The maximum number of nodes than we can get in order to have peak irradiance (assuming 1 μm beam radius) around 100 $GW.cm^{-2}$ (which is enough to enable TPA and SA in the materials that we have used in the experiment) is roughly N= 4500. The pulse width increases from 50 femtosecond (fs) to 141 picosecond (ps) for each channel, based on the Fourier transform relationship. Note that the number of comb teeth for the mentioned bandwidth, $\Delta\lambda = 30$ nm, is very large. Thus, theoretically dividing the spectral bandwidth into 4500 nodes would not be a fundamental problem (number of comb teeth= $\frac{\Delta\nu}{\text{Rep rate}} = \frac{14\times10^{12}\,\text{Hz}}{1000\ \text{Hz}} \approx 14 \times 10^{9}$). For a million nodes graph size, N=10^6, the pulse width expands to 30 ns and the peak irradiance drops to 2 $MW.cm^{-2}$ (assuming 1 μm beam radius) which is not enough to enable SA and TPA behavior for most known materials in nature. Theoretically, if we can expand the spectral bandwidth of the laser source, we would in principle be able to increase the number of nodes.

Supplementary References

1. Wainwright, M, J. & Jordan, M. I. *Graphical Models, Exponential Families, and Variational Inference*. (Now Publishers, 2008).

2. Cheung, S., Su, T., Okamoto, K. & Yoo, S. J. B. Ultra-compact silicon photonic 512 × 512 25 GHz arrayed waveguide grating router. *IEEE J. Sel. Top. Quantum Elect*. **20,** Issue: 4 (2014).

3. Kamei, S., Ishii, M., Kitagawa, I., Itoh, M. & Hibino, Y. Very low crosstalk arrayed-waveguide grating multi/demultiplexer using cascade connection technique. *IEEE Electronics Letters*. **36,** 823 - 824 (2000).

4. Kamei, S., Ishii, M., Kitagawa, I., Itoh, M. & Hibino, Y. 64-channel ultra-low crosstalk arrayed-waveguide grating multi/demultiplexer module using cascade connection technique. *IEEE Electronics Letters*. **39,** 81 - 82 (2003).

5. Wang, Z. et al. A III-V-on-Si ultra-dense comb laser. Light: Science & Applications. http://dx.doi.org/10.1038/lsa.2016.260 (2017).

6. Klenner, A. et al. Gigahertz frequency comb offset stabilization based on supercontinuum generation in silicon nitride waveguides. *Opt. Express* **24 (10),** 11043-11053 (2016).

7. Ozdur, I. et al. Semiconductor based optical frequency comb source with optical linewidth ≪1 kHz. *IEEE. LEOS Conf. Proc.* 491-492 (2009).

8. Diddams, S. A., Hollberg, L. & Mbele, V. Molecular fingerprinting with the resolved modes of a femtosecond laser frequency comb. *Nature* **445,** 627-630 (2007).

9. Bartels, A., Oates, C. W., Hollberg, L. & Diddams, S. A. Stabilization of femtosecond laser frequency combs with subhertz residual linewidths. *Opt. Letter* **29(10),** 1081-1083 (2004).

10. Shirasaki, M. Large angular dispersion by a virtually imaged phased array and its application to a wavelength demultiplexer. *Opt. Letter* **21(5),** 366-368 (1996).
11. Jeffers, J., Reinders, J. & Sodani, A. *Intel Xeon Phi Processor High Performance Programming: Knights Landing Edition*. (Morgan Kaufmann, Burlington, 2016).
12. Jouppi, N. P. et al. In-datacenter performance analysis of a tensor processing unit™. The 44th international symposium on computer architecture (ISCA), Toronto, Canada. https://arxiv.org/abs/1704.04760 (2017).
13. Caulfield, H. J. & Dolev, S. Why future supercomputing requires optics. *Nat. photon.* **4**, 261–263 (2010).
14. Heddeghem, W. V. et al. Trends in worldwide ICT electricity consumption from 2007 to 2012. *Comput. Commun.* **50,** 64–76 (2014).
15. Miller, D. A. B. Device requirements for optical interconnects to silicon chips. *Proc. IEEE.* **97 (7),** 1166–1185 (2009).
16. Miller, D. A. B. Attojoule Optoelectronics for Low-Energy Information Processing and Communications. *J. Lightwave. Tech.* **35 (3),** 343-393 (2017).
17. Mattevi, C., Kim, K. & Chhowalla, M. A review of chemical vapour deposition of graphene on copper. *Jour. Mater. Chem.* **21,** 3324–3334 (2011).
18. Kaplas, T. & Kuzhir, P. Ultra-thin graphitic film: synthesis and physical properties. *Nanoscale. Res. Let.* **11:54,** 1–6 (2016).
19. Jawhari, T., Roid, A. & Casado, J. Raman spectroscopic characterization of some commercially available carbon black materials. *Carbon.* **33,** 1561-1565 (1995).
20. Ferrari, A. C. & Robertson, J. Raman spectroscopy of amorphous, nanostructured, diamond-like carbon. *Phil. Trans. Roy. Soc.* **362,** 2477–2512 (2004).

21. Mak, K. F., Ju, L., Wang, F. & Heinz, T. F. Optical spectroscopy of graphene: From the far infrared to the ultraviolet. *Solid state Comm.* **152,** 1341–1349 (2012).

22. Kostecki, R. et al. Surface studies of carbon films from pyrolyzed photoresist. *Thin solid Films.* **396,** 36–43 (2001).

23. Kaplas, T. & Kuzhir, P. Ultra-thin pyrocarbon films as a versatile coating material. *Nanoscale. Res. Let.* **12,** 1–6 (2017).

24. Barlow, S. et al. Polymethine materials with solid-state third-order optical susceptibilities suitable for all-optical signal-processing applications. *Mater. Horiz.* **1**, 577–581 (2014).

25. Anderson, H. L., Martin, S. J. & Bradley, D. D. C. Synthesis and third-order nonlinear optical properties of a conjugated porphyrin polymer. *Angew. Chem. Int. Ed.* **33,** 655–657 (1994).

26. Singer, K. D. et al. Electro-optic phase modulation and optical second-harmonic generation in corona-poled polymer films. *Appl. Phys. Lett.* **53,** 1800– 1802 (1988).

27. Pascal, S. et al. Symmetry loss of heptamethine cyanines: an example of dipole generation by ion-pairing effect. *Proc. of SPIE.* **8622,** 86220F-1 (2013).
